# Upregulated lncRNA‐NEF predicts recurrence and poor treatment outcomes of ankylosing spondylitis

**DOI:** 10.1002/iid3.627

**Published:** 2022-07-12

**Authors:** Dapeng Han, Guilin Ouyang, Peijun Pan, Yuan Yuan

**Affiliations:** ^1^ Department of Orthopaedics Shanghai Guanghua Hospital of Integrated Traditional Chinese and Western Medicine Shanghai China; ^2^ Department of the Health and Nursing Shanghai Nanhu Vocational and Technical College Shanghai China

**Keywords:** ankylosing spondylitis, lncRNA‐NEF, recurrence, synovial fluid

## Abstract

**Introduction:**

Osteoporosis is related to lncRNA‐neighboring enhancer of FOXA2 (NEF) and inversely correlated to ankylosing spondylitis (AS), implying that lncRNA‐NEF might also relate to AS. Thus, the study was carried out to investigate the involvement of lncRNA‐NEF in AS.

**Methods:**

The study included 60 AS patients and 60 healthy controls. LncRNA‐NEF expression in synovial fluid samples was analyzed by reverse transcription quantitative real‐time polymerase chain reaction. Disease activity of the 60 AS patients was determined using the Ankylosing Spondylitis Disease Activity Score (ASDAS) 1–4 and Bath Ankylosing Spondylitis Disease Activity Index (BASDAI). Western blot was carried out to investigate the effects of lncRNA‐NEF on inflammatory factors in human fibroblast‐like synovial (HFLS) cells. A 3‐year follow‐up was performed to analyze the role of lncRNA‐NEF in the prediction of the recurrence of AS.

**Results:**

Our study observed that lncRNA‐NEF expression was upregulated in synovial fluid of AS patients and significantly correlated with the ASDAS 1–4, BASDAI, erythrocyte sedimentation rate (ESR), and C‐reactive protein level (*p* < .05). Treatment with nonsteroidal anti‐inflammatory drugs significantly downregulated lncRNA‐NEF expression (*p* < .01). A 3‐year follow‐up showed that patients with high lncRNA‐NEF levels had a high recurrence rate (hazard ratio = 2.266). In addition, lncRNA‐NEF was found to regulate the expression of inflammatory factors in HFLS cells.

**Conclusions:**

Therefore, lncRNA‐NEF upregulation can predict recurrence and poor treatment outcomes of AS and has a great potential to serve as a predictive biomarker factor for the recurrent AS.

## Graphical abstract

LncRNA‐NEF expression was upregulated in synovial fluid of ankylosing spondylitis (AS) patients and significantly correlated with the Ankylosing Spondylitis Disease Activity Score 1–4, BASDAI, erythrocyte sedimentation rate and C‐reactive protein level. Treatment with nonsteroidal anti‐inflammatory drugs significantly downregulated lncRNA‐NEF expression. LncRNA‐NEF upregulation can predict recurrence and poor treatment outcomes of AS.

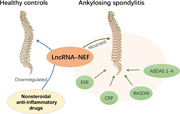

## 1 BACKGROUND

As a rare type of arthritis, ankylosing spondylitis (AS) mainly affects the lower back, spine, and sacroiliac joints.^1^ Patients with AS usually suffer from severe pain, stiffness, and loss of mobility.^2^ AS is a lifelong clinical disorder with no curable treatment available currently.^3^ The available treatments are intended only to alleviate its symptoms and slow down the process of spinal stiffness and spinal fusion.^4^ Despite the efforts made for AS treatment, AS is still not fully reversible, especially for patients with advanced lesions.^5^ Therefore, novel therapeutic targets and approaches to predict recurrence and poor treatment outcomes are urgently needed.

Recent progress in understanding the molecular pathogenesis of AS has revealed that many molecules are involved in AS.^6^, ^7^ Understanding the functions of these molecular factors, such as HLA‐B27 gene,^8^ provides novel insights into the development of anti‐AS therapies.^9^ Besides proteins, long noncoding RNAs (lncRNAs) are also involved in human diseases by regulating disease‐related gene expression.^10^, ^11^ In many human diseases, including AS, lncRNAs may directly regulate the expression of protein‐coding genes by binding to their promoter region or serve as endogenous competing RNA for miRNAs to indirectly regulate downstream protein‐coding genes, thereby participating in disease progression.^10^, ^11^ It has been reported that AS is closely correlated to osteoporosis.^12^ AS causes systemic inflammation, which is also common in osteoporosis. In effect, osteoporosis is a common complication in patients with AS.^12^ LncRNA‐NEF is known to participate in osteoporosis,^13^ suggesting its possible involvement in AS. Therefore, this study was carried out to investigate the involvement of lncRNA‐NEF in AS.

## 2 METHODS

### 2.1 AS patients and healthy controls

A total of 60 AS patients (37 males and 23 females, aged 25–47 years, mean ± SD: 30.9 ± 4.9 years) and 60 healthy controls (37 males and 23 females, aged 24–47 years, mean ± SD: 31.0 ± 4.7 years) who admitted to our hospital between March 2015 and March 2016 were enrolled in the study (Table 1). All AS patients were diagnosed by X‐ray based on the inflammation of the sacroiliac joint between the sacrum and the ilium (arthritis) for the first time with no prior therapy.^14^ In addition, all patients showed inflammatory back pain, and those with other severe clinical disorders were excluded. The 60 healthy controls received systemic physiological exams at the Health Center of our hospital and had normal physiological functions without using non‐steroidal anti‐infiammatory drugs. The mean C‐reactive protein (CRP) value was 1.9 (0.7–2.9) mg/L for the healthy controls and 45.2 (10.7–96.9) mg/L for the AS patients. The mean estrogen receptor (ESR) value (in the first hour) was 11.3 (3.1–19.7) mm for the healthy controls and 79.2 (42.1–119.1) mm for the AS patients. All participants signed written informed consent.

**Table 1:** Clinical features of AS and control groups

Characteristic	AS patients (*n* = 60)	Healthy controls (*n* = 60)
Gender		
Male	37	37
Female	23	23
Age (years)	25–47 (30.9 ± 4.9)	24–47 (31.0 ± 4.7)
CRP (mg/L)	45.2 (10.7–‐96.9)	1.9 (0.7–2.9)
ESR (mm)	79.2 (42.1–119.1)	11.3 (3.1–19.7)

Abbreviations: AS, ankylosing spondylitis; CRP, C‐reactive protein; ESR, estrogen receptor.

### 2.2 Synovial fluid

A total of 2 ml of synovial fluid was extracted from all affected sites (38 cases of the joint between the base of the spine and pelvis and 22 cases of vertebrae in the lower back) of each AS patient and the corresponding sites of each healthy control. All fresh synovial fluid samples were stored in liquid nitrogen before use.

### 2.3 Determination of disease activity

Disease activity was determined using Ankylosing Spondylitis Disease Activity Score (ASDAS) 1–4 and Bath Ankylosing Spondylitis Disease Activity Index (BASDAI). ESR and CRP levels in synovial fluid samples of AS patients were determined using the ESR Alpha ELISA Kit (ab128499; Abcam) and CRP ELSA Kit (CRP) (ab99995; Abcam), respectively. All steps were completed following the manufacturers’ instructions.

### 2.4 Treatment and follow‐up

AS patients were treated for 3 months by oral administration of nonsteroidal anti‐inflammatory drugs, including naproxen (oral, 0.25 g, two times a day) and indomethacin (oral, 25−50 mg, three to four times a day).^15^, ^16^, ^17^ Synovial fluid (2 ml) was extracted from the affected sites of each AS patient after the above treatment. After discharge, patients were followed up by telephone and/or outpatient visits every month for 3 years, and the recurrence of AS (significant pain, stiffness, and deterioration of ASDAS and BASDAI) was recorded. ASDAS included four scores based on low back pain, morning stiffness time, peripheral joint swelling, and pain using a 10 cm visual simulation scale (blood vessel), where 0 for no discomfort and 10 for the most severe discomfort. In addition, ASDAS‐CRP index was calculated as 0.121 × low back pain + 0.058 × morning stiffness duration + 0.11 × overall evaluation of patients + 0.073 × peripheral pain relief/swelling + 0.579 × Ln (CRP + 1) and ASDAS‐ESR index was calculated as 0.079 × low back pain + 0.069 × morning stiffness duration + 0.113 × overall evaluation of patients + 0.086 × peripheral joint pain/swelling + 0.293 × ESR. BASDAI was defined as the average score of fatigue, spinal pain, joint pain, tendinitis, and spondylitis. Each item was scored based on the visual analogue scale  (0−10) score of patients' self‐evaluation. Spondylitis score was defined as the average morning stiffness score and morning stiffness time score.

### 2.5 Cell extraction and culture

Human fibroblast‐like synovial (HFLS) cells were extracted from AS patients for in vitro experiments. Firstly, the synovial tissues of AS patients were cleaned with phosphate‐buffered saline (PBS), sliced, and collected into the digestion bottles. The tissues were digested with collagenase (Gibco) for 1h and centrifuged. The precipitated cells were resuspended in dulbecco's modified eagle's medium (DMEM; Gibco) containing 20% fetal bovine serum (FBS; Gibco) and cultured at 37℃ for 24 h in an incubator with 5% CO_2_. After adherent to the surface, cells were washed with PBS and cultured in DMEM containing 10% FBS for future experiments.

### 2.6 Cell transfection

LncRNA‐NEF overexpression plasmid, small interference sequence (si‐NEF), and their negative controls (pcDNA or si‐con) were provided by Vigene Biosciences. HFLS cell transfection was performed using Lipofectamine 2000 (Invitrogen).

### 2.7 RNA preparation and RT‐qPCR

Synovial fluid samples were subjected to RNA isolation using RNAzol reagent (Sigma‐Aldrich). After digestion with DNase I (Invitrogen) to completely remove genomic DNA, RNA samples were reverse‐transcribed into complementary DNA (cDNA) samples. With cDNA samples as templates, lncRNA‐NEF levels were measured by qPCR using QuantiFast SYBR Green PCR Kits (Qiagen) with 18S ribosomal ribonucleic acid (rRNA) as the internal control. All PCR reactions were performed in three replicates. Relative gene expression was calculated using $2^{‐∆∆C_{t}}$. Primer sequences were 5′‐CTGCCGTCTTAAACCAACCC‐3′ and 5′‐GCCCAAACAGCTCCTCAATT‐3′ for lncRNA‐NEF, and 5′‐AGGCGCGCAAATTACCCAATCC‐3′ and 5′‐GCCCTCCAATTGTTCCTCGTTAAG‐3′ for 18S rRNA.

### 2.8 Western blot

HFLS cells were lysed with RIPA lysate (Beyotime). Total proteins were separated by sodium dodecyl sulfate‐polyacrylamide gel electrophoresis and transferred onto polyvinylidene fluoride membranes (Millipore). The membranes were incubated with 5% skimmed milk for 1 h, and incubated overnight at 4℃ with primary antibodies against interleukin‐1β (IL‐1β) (1:1000, ab216995; Abcam), interleukin‐6 (IL‐6) (1:1000, ab233706; Abcam), tumour necrosis factor‐α (TNF‐α) (1:1000, ab215188; Abcam), and GAPDH (1:1000, ab8245; Abcam). After washing, the membranes were incubated with a secondary antibody (1:5,000; Biotech) at room temperature for 4 h. The signals were developed using Pierce ECL Western Blot Substrate (Thermo Fisher Scientific), imaged, and analyzed with GAPDH as the control using ImageJ software.

### 2.9 Statistical analysis

Statistical analysis was performed using SPSS 19.0 software (SPSS). The data were expressed as mean ± standard deviation (SD). The differences between two groups were evaluated using Student's *t*‐test and among multiple groups were analyzed using one‐way analysis of variance. *χ* ^2^ test was used to analyze the correlations of lncRNA‐NEF levels with ASDAS 1–4, BASDAI, and the levels of ESR and CRP in synovial fluid samples by taking age and gender as covariates. *p* < .05 was considered statistically significant.

## 3 RESULTS

### 3.1 LncRNA‐NEF expression in synovial fluid of AS patients and its predictive value for recurrence

**Figure 1:**
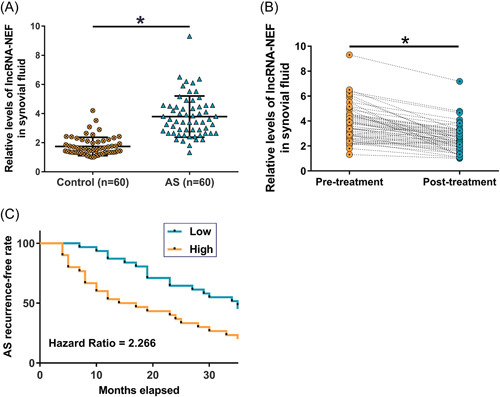
LncRNA‐NEF expression in synovial fluid of ankylosing spondylitis (AS) patients and its predictive value for recurrence LncRNA‐NEF levels in synovial fluid samples from AS patients (*n* = 60) and healthy controls (*n* = 60) were measured by reverse transcription quantitative real‐time polymerase chain reaction **(**RT‐qPCR) (A). LncRNA‐NEF levels in synovial fluid samples from the 60 AS patients before and after treatment with nonsteroidal anti‐inflammatory drugs were measured by RT‐qPCR and compared (B). The 60 AS patients were divided into high and low lncRNA‐NEF level groups (*n* = 30) with the median pretreatment lncRNA‐NEF level as the cutoff value. Based on the 3 years’ follow‐up data, AS recurrence‐free curves were plotted for both groups and compared using the log‐rank test (C). *p < .05.

LncRNA‐NEF expression in synovial fluid samples from AS patients (*n* = 60) and healthy controls (*n* = 60) was measured by RT‐qPCR. LncRNA‐NEF expression was significantly higher in the AS group than in the control group (Figure 1A, 2.12‐fold, *p* < .05), suggesting that lncRNA‐NEF might involve in AS development. LncRNA‐NEF expression in synovial fluid samples from the 60 AS patients before and after treatment was measured by RT‐qPCR. The results showed that treatment with nonsteroidal anti‐inflammatory significantly decreased lncRNA‐NEF expression (Figures 1B, 1.42‐fold, *p* < .05). Therefore, measuring lncRNA‐NEF expression in synovial fluid might reflect the recovery of AS. The 60 AS patients were divided into high and low lncRNA‐NEF level groups (*n* = 30) with the median pretreatment lncRNA‐NEF level as the cutoff. Recurrence was observed in 24 cases and 17 cases in high and low lncRNA‐NEF level groups, respectively. AS recurrence‐free curves were plotted for both high and low lncRNA‐NEF level groups. The recurrence rate of AS was significantly lower in patients in the low lncRNA‐NEF level group than in the high lncRNA‐NEF level group (Figure 1C, hazard ratio = 2.266), suggesting that lncRNA‐NEF might serve as a prognostic biomarker for AS.

### 3.2 LncRNA‐NEF expression was correlated with ASDAS 1–4, BASDAI, and the levels of ESR and CRP

*χ* ^2^ test was used to analyze the correlations of lncRNA‐NEF expression with ASDAS 1–4, BASDAI, and the levels of ESR and CRP in synovial fluid samples. It was observed that lncRNA‐NEF expression in synovial fluid samples was significantly correlated with ASDAS 1–4, BASDAI, and the levels of ESR and CRP, showing positive linear correlations (Table 2, all *p* < .01). Moreover, treatment with nonsteroidal anti‐inflammatory drugs significantly changed ASDAS 1–4, BASDAI, and the levels of ESR and CRP (all *p* < .01).

**Table 2:** Correlations between levels of lncRNA‐NEF and ASDAS 1–4, BASDAI, and levels of ESR and CRP in synovial fluid samples

Markers	ESR		CRP		lncRNA‐NEF	
	*R* ^2^	*p* value	*R* ^2^	*p* value	*R* ^2^	*p* value
ASDAS 1	0.66	<.01	0.71	<.01	0.62	<.01
ASDAS 2	0.67	<.01	0.70	<.01	0.61	<.01
ASDAS 3	0.67	<.01	0.72	<.01	0.63	<.01
ASDAS 4	0.68	<.01	0.73	<.01	0.62	<.01
BASDAI	0.72	<.01	0.73	<.01	0.66	<.01
ESR	1.00	<.01	0.89	<.01	0.59	<.01
CRP	0.89	<.01	1.00	<.01	0.60	<.01

Abbreviations: ASDAS, Ankylosing Spondylitis Disease Activity Score; CRP, C‐reactive protein; ESR, estrogen receptor; lncRNA, long noncoding RNAs.

### 3.3 LncRNA‐NEF regulated the expression of inflammatory factors in HFLS

**Figure 2:**
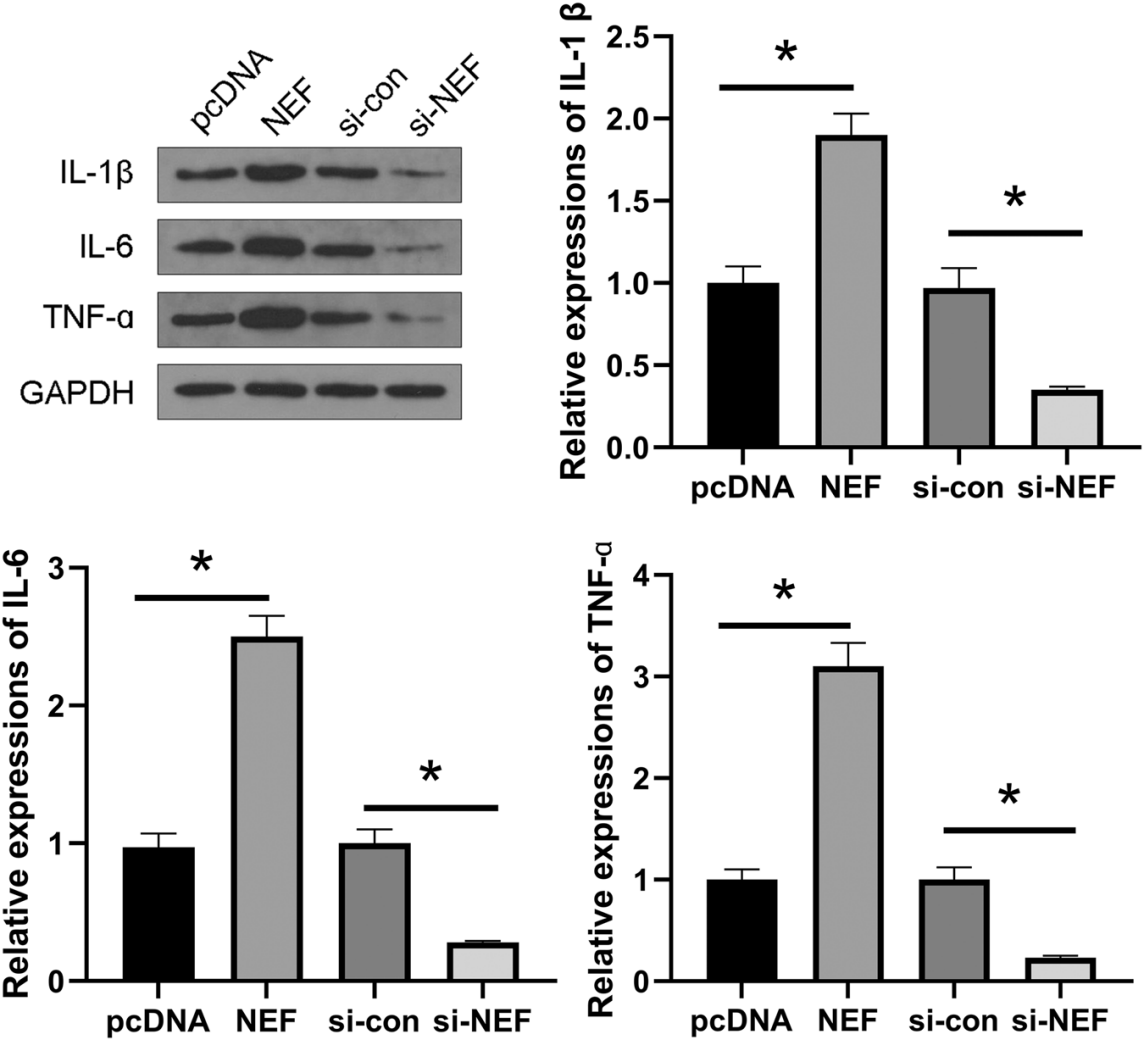
NEF regulated the expression of inflammatory factors in human fibroblast‐like synovial (HFLS). HFLS cells were obtained from ankylosing spondylitis patients and transfected with NEF overexpression plasmid, si‐NEF, or their negative controls (pcDNA or si‐con). The protein levels of interleukin‐1β (IL‐1β), interleukin‐6 (IL‐6), and tumor necrosis factor‐α (TNF‐α) in these cells were detected by Western blot. LncRNA‐NEF overexpression promoted the expression of inflammatory factors, while lncRNA‐NEF knockdown inhibited the expression of inflammatory factors. *p < .05.

HFLS cells from AS patients were obtained and transfected with lncRNA‐NEF overexpression plasmid, si‐NEF, or their negative controls (pcDNA or si‐con). The protein levels of IL‐1β, IL‐6, and TNF‐α were detected using Western blot. It was observed that lncRNA‐NEF overexpression significantly increased the protein levels of IL‐1β, IL‐6, and TNF‐α while lncRNA‐NEF downregulation significantly decreased the protein levels of IL‐1β, IL‐6, and TNF‐α (Figure 2, all *p* < .05). These results suggested that lncRNA‐NEF might regulate the expression of inflammatory factors in HFLS.

## 4 DISCUSSION

This study analyzed the involvement of lncRNA‐NEF in AS and found that lncRNA‐NEF was overexpressed in AS and a high lncRNA‐NEF level might predict a high AS recurrence rate.

The development and progression of AS require the involvement of many dysregulated lncRNAs,^18^ some of which have been proven to be critical players in AS. For instance, lncRNA TUG1 was downregulated in AS and predicted the disease activity and treatment course.^19^ LncRNA MEG3 was downregulated in AS and played an anti‐inflammatory role in AS partially by targeting miR‐146a.^20^ LncRNA‐NEF was downregulated in postmenopausal osteoporosis and related to the course of its treatment and recurrence.^13^ It has been reported that AS is inversely related to osteoporosis.^12^ Therefore, we hypothesized that lncRNA‐NEF might also participate in AS. Our results showed that lncRNA‐NEF level was increased in AS and significantly decreased after treatment with nonsteroidal anti‐inflammatory drugs, suggesting that lncRNA‐NEF might participate in AS and reflect the treatment effect on AS. However, the function of lncRNA‐NEF in AS development and its underlying molecular mechanism remains unclear and needs to be further explored.

The recurrence rate of AS patients is high even after active treatment^20^ because the currently available treatments cannot relieve the symptoms. Besides, AS is unlikely curable in the near future. Therefore, accurately predicting AS recurrence may help improve the quality of life of AS patients by guiding the development of treatment programs. Our study showed that a high lncRNA‐NEF level before treatment was closely correlated with the high recurrence rate of AS. Therefore, lncRNA‐NEF has a great potential to serve as a predictive biomarker for recurrent AS. However, due to the limited clinical sample size, its prediction accuracy needs to be further tested in a multicenter cohort with a large sample size. In this study, lncRNA‐NEF positively regulated the expression of IL‐1β, IL‐6, and TNF‐α in HFLS. Previous studies have shown that lncRNAs may interact with miRNAs to regulate inflammation in AS. For instance, lncRNA MEG3 targets miR‐146a to inhibit the inflammatory response in AS.^20^ LncRNA H19 interacts with miR675‐5p/miR22‐5p to affect VDR expression, thereby increasing the secretion of IL‐17A/IL‐23 in AS.^21^ Therefore, lncRNA‐NEF may also regulate the expression of IL‐1β, IL‐6, and TNF‐α through certain miRNAs. However, the mechanism of lncRNA‐NEF action remains to be studied.

## 5 CONCLUSION

LncRNA‐NEF is overexpressed in AS, and a high lncRNA‐NEF level may predict a high posttreatment recurrence rate of AS.

## AUTHOR CONTRIBUTIONS

**Dapeng Han** and **Yuan Yuan**: Concept, manuscript writing; editing and review. **Guilin Ouyang** and **Peijun Pan**: Data collection and analysis; manuscript preparation. All authors have read and approved the submission of the manuscript.

## CONFLICT OF INTEREST

The authors declare no conflict of interest.

## ETHICS STATEMENT

All patients signed the written informed consent. All procedures were approved by the Ethics Committee of Shanghai Nanhu Vocational and Technical College (IRB approval No. 64#322) and completed in keeping with the standards set out in the Announcement of Helsinki and laboratory guidelines of research in China.

## Supporting information

Supporting information.

Supporting information.

Supporting information.

Supporting information.

## Data Availability

The analyzed data sets generated during the study are available from the corresponding author on reasonable request.

## References

[1] Taurog JD , Chhabra A , Colbert RA . Ankylosing spondylitis and axial spondyloarthritis. N Engl J Med. 2016;374(26):2563‐2574. 27355535 10.1056/NEJMra1406182

[2] Zochling J . Measures of symptoms and disease status in ankylosing spondylitis: Ankylosing Spondylitis Disease Activity Score (ASDAS), Ankylosing Spondylitis Quality of Life Scale (ASQoL), Bath Ankylosing Spondylitis Disease Activity Index (BASDAI), Bath Ankylosing Spondylitis Functional Index (BASFI), Bath Ankylosing Spondylitis Global Score (BAS‐G), Bath Ankylosing Spondylitis Metrology Index (BASMI), Dougados Functional Index (DFI), and Health Assessment Questionnaire for the Spondylarthropathies (HAQ‐S). Arthritis Care Res. 2011;63(suppl 11):S47‐S58. 10.1002/acr.20575 22588768

[3] Smolen JS , Braun J , Dougados M , et al. Treating spondyloarthritis, including ankylosing spondylitis and psoriatic arthritis, to target: recommendations of an international task force. Ann Rheum Dis. 2014;73(1):6‐16. 23749611 10.1136/annrheumdis-2013-203419 PMC3888616

[4] Vastesaeger N , van der Heijde D , Inman RD , et al. Predicting the outcome of ankylosing spondylitis therapy. Ann Rheum Dis. 2011;70(6):973‐981. 21402563 10.1136/ard.2010.147744 PMC3086037

[5] Patel P , Hussain H , Fahey J . Delayed diagnosis of ankylosing spondylitis: a missed opportunity? Cureus. 2019;11(9):e5723. 31720191 10.7759/cureus.5723 PMC6823060

[6] Alvarez‐Navarro C , Lopez de Castro JA . ERAP1 structure, function and pathogenetic role in ankylosing spondylitis and other MHC‐associated diseases. Mol Immunol. 2014;57(1):12‐21. 23916068 10.1016/j.molimm.2013.06.012

[7] Brown MA , Kenna T , Wordsworth BP . Genetics of ankylosing spondylitis‐insights into pathogenesis. Nat Rev Rheumatol. 2016;12(2):81‐91. 26439405 10.1038/nrrheum.2015.133

[8] Chen B , Li J , He C , et al. Role of HLA‐B27 in the pathogenesis of ankylosing spondylitis (review). Mol Med Rep. 2017;15(4):1943‐1951. 28259985 10.3892/mmr.2017.6248 PMC5364987

[9] Baeten D , Baraliakos X , Braun J , et al. Anti‐interleukin‐17A monoclonal antibody secukinumab in treatment of ankylosing spondylitis: a randomised, double‐blind, placebo‐controlled trial. Lancet. 2013;382(9906):1705‐1713. 24035250 10.1016/S0140-6736(13)61134-4

[10] Wapinski O , Chang HY . Long noncoding RNAs and human disease. Trends Cell Biol. 2011;21(6):354‐361. 21550244 10.1016/j.tcb.2011.04.001

[11] Lalevee S , Feil R . Long noncoding RNAs in human disease: emerging mechanisms and therapeutic strategies. Epigenomics. 2015;7(6):877‐879. 26418705 10.2217/epi.15.55

[12] Hinze AM , Louie GH . Osteoporosis management in ankylosing spondylitis. Curr Treatm Opt Rheumatol. 2016;2(4):271‐282. 28620575 10.1007/s40674-016-0055-6 PMC5467452

[13] Ma X , Guo Z , Gao W , et al. LncRNA‐NEF is downregulated in postmenopausal osteoporosis and is related to course of treatment and recurrence. J Int Med Res. 2019;47(7):3299‐3306. 31220986 10.1177/0300060519847854 PMC6683934

[14] Linden SVD , Valkenburg HA , Cats A . Evaluation of diagnostic criteria for ankylosing spondylitis. Arthritis Rheumatol. 1984;27(4):361‐368. 10.1002/art.1780270401 6231933

[15] Kroon FP , van der Burg LR , Ramiro S , et al. Non‐steroidal anti‐inflammatory drugs (NSAIDs) for axial spondyloarthritis (ankylosing spondylitis and non‐radiographic axial spondyloarthritis). Cochrane Database Syst Rev. 2015;7:CD010952. 10.1002/14651858.CD010952.pub2 PMC8942090 26186173

[16] Zochling J , Bohl‐Bühler M , Baraliakos X , Feldtkeller E , Braun J . Nonsteroidal anti‐inflammatory drug use in ankylosing spondylitis—a population‐based survey. Clin Rheumatol. 2006;25(6):794‐800. 16528455 10.1007/s10067-005-0132-y

[17] Dooley M , Spencer C , Dunn C . Aceclofenac: a reappraisal of its use in the management of pain and rheumatic disease. Drugs. 2001;61(9):1351‐1378. 11511027 10.2165/00003495-200161090-00012

[18] Zhang C , Wang C , Jia Z , et al. Differentially expressed mRNAs, lncRNAs, and miRNAs with associated co‐expression and ceRNA networks in ankylosing spondylitis. Oncotarget. 2017;8(69):113543‐113557. 29371928 10.18632/oncotarget.22708 PMC5768345

[19] Lan X , Ma H , Zhang Z , et al. Downregulation of lncRNA TUG1 is involved in ankylosing spondylitis and is related to disease activity and course of treatment. Biosci Trends. 2018;12(4):389‐394. 30146549 10.5582/bst.2018.01117

[20] Li Y , Zhang S , Zhang C , Wang M . LncRNA MEG3 inhibits the inflammatory response of ankylosing spondylitis by targeting miR‐146a. Mol Cell Biochem. 2020;466(1‐2):17‐24. 31894531 10.1007/s11010-019-03681-x

[21] Zhang X , Ji S , Cai G , et al. H19 increases IL‐17A/IL‐23 releases via regulating VDR by interacting with miR675‐5p/miR22‐5p in ankylosing spondylitis. Mol Ther Nucleic Acids. 2020;19:393‐404. 31887550 10.1016/j.omtn.2019.11.025 PMC6938967

