# Supplementary figures and images for: Upregulated lncRNA‐NEF predicts recurrence and poor treatment outcomes of ankylosing spondylitis
Source: Immun Inflamm Dis. 2022 Jul 12;10(8):e627. doi: 10.1002/iid3.627 (PMC9274798; doi:10.1002/iid3.627)

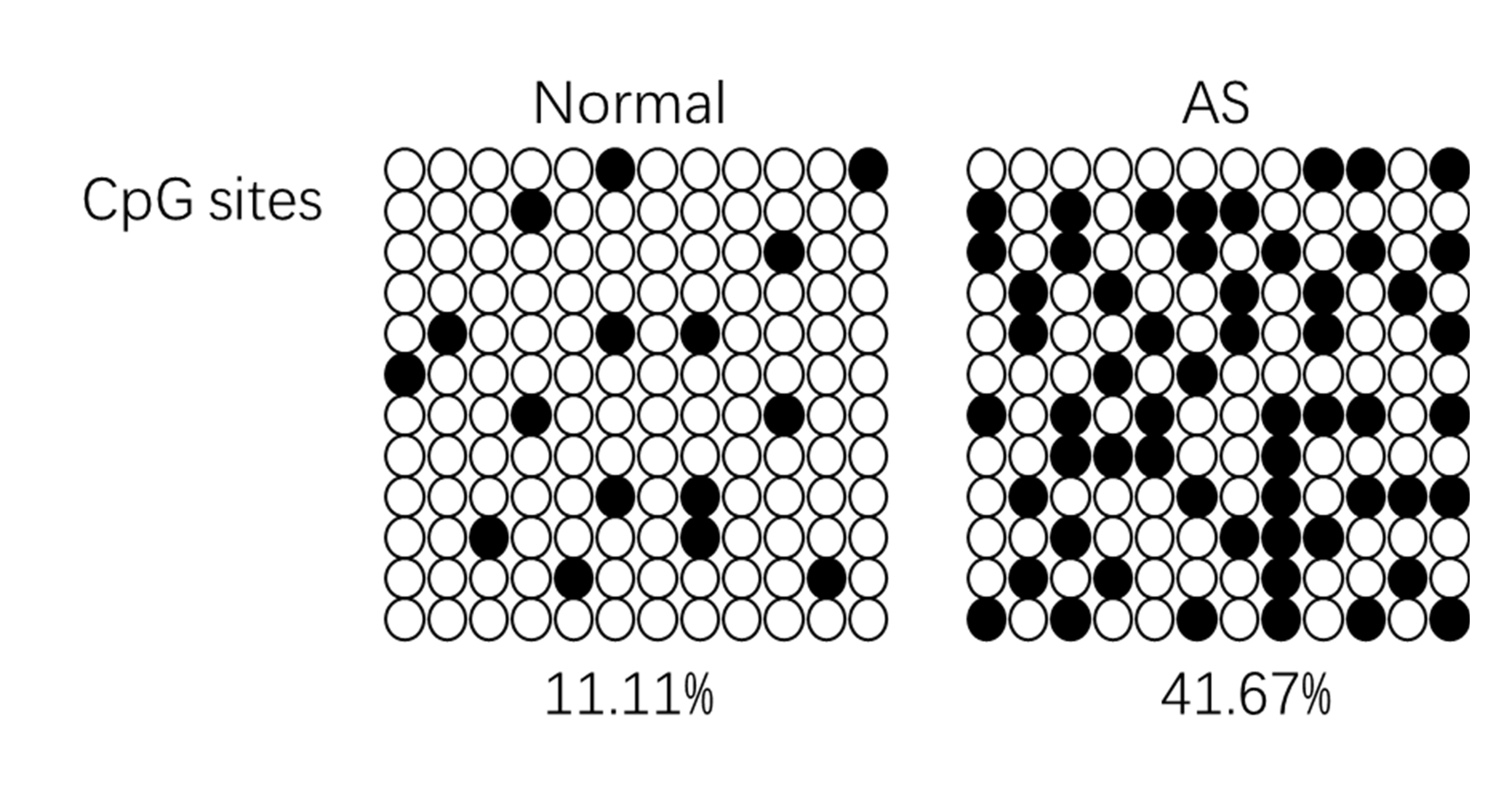

Supplement: Supplementary file 1 — Supporting information. [file IID3-10-e627-s001.tif]

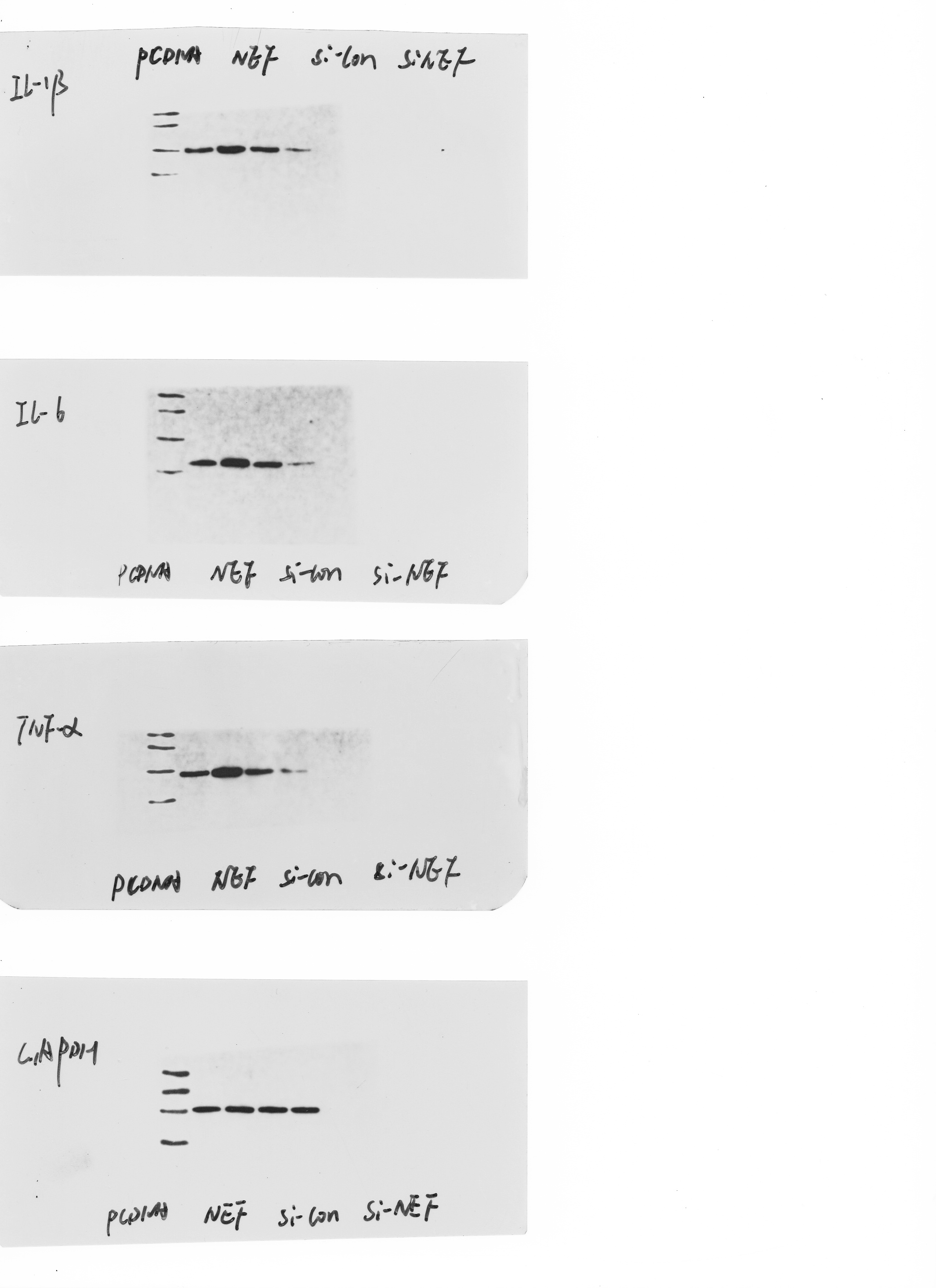

Supplement: Supplementary file 2 — Supporting information. [file IID3-10-e627-s002.tif]
