# Supplementary material for: Upregulated lncRNA‐NEF predicts recurrence and poor treatment outcomes of ankylosing spondylitis
Source: Immun Inflamm Dis. 2022 Jul 12;10(8):e627. doi: 10.1002/iid3.627 (PMC9274798; doi:10.1002/iid3.627)
Supplement: Supplementary file 3 — Supporting information. [file IID3-10-e627-s004.docx]

Table 1 Clinical features of AS and control groups.

| Characteristic | AS patients（n=60） | Healthy controls (n=60) |
| --- | --- | --- |
| Gender |  |  |
| Male | 37 | 37 |
| Female | 23 | 23 |
| Age (years) | 25-47 (30.9 ± 4.9) | 24-47 (31.0 ± 4.7) |
| CRP (mg/L) | 45.2 (10.7-96.9) | 1.9 (0.7-2.9) |
| ESR (mm) | 79.2 (42.1-119.1) | 11.3 (3.1-19.7) |
