# Supplementary material for: Upregulated lncRNA‐NEF predicts recurrence and poor treatment outcomes of ankylosing spondylitis
Source: Immun Inflamm Dis. 2022 Jul 12;10(8):e627. doi: 10.1002/iid3.627 (PMC9274798; doi:10.1002/iid3.627)
Supplement: Supplementary file 4 — Supporting information. [file IID3-10-e627-s003.docx]

**Table 2 Correlations between levels of lncRNA-NEF and ASDAS 1-4, BASDAI, and levels of ESR and CRP in synovial fluid samples.**

| Markers | ESR | | CRP | | lncRNA-NEF | |
| --- | --- | --- | --- | --- | --- | --- |
|  | R square | p value | R square | p value | R square | p value |
| ASDAS 1 | 0.66 | <0.01 | 0.71 | <0.01 | 0.62 | <0.01 |
| ASDAS 2 | 0.67 | <0.01 | 0.70 | <0.01 | 0.61 | <0.01 |
| ASDAS 3 | 0.67 | <0.01 | 0.72 | <0.01 | 0.63 | <0.01 |
| ASDAS 4 | 0.68 | <0.01 | 0.73 | <0.01 | 0.62 | <0.01 |
| BASDAI | 0.72 | <0.01 | 0.73 | <0.01 | 0.66 | <0.01 |
| ESR | 1.00 | <0.01 | 0.89 | <0.01 | 0.59 | <0.01 |
| CRP | 0.89 | <0.01 | 1.00 | <0.01 | 0.60 | <0.01 |
